# Selinexor enhances temozolomide efficacy in glioblastoma through modulation of multiple resistance-associated mechanisms

**DOI:** 10.3389/fonc.2026.1916260

**Published:** 2026-09-28

**Authors:** Beyza Ozturk, Asuman Celebi, Deniz Kilic, Turker Kilic, Timucin Avsar

**Affiliations:** 1 Neurooncology Laboratory, Neuroscience PhD Program, Graduate School, Bahçeşehir University, İstanbul, Türkiye; 2 Department of Medical Biology, Faculty of Medicine, Bahçeşehir University, İstanbul, Türkiye; 3 Department of Neurosurgery, School of Medicine, Bahçeşehir University, Goztepe Medical Park Hospital, Istanbul, Türkiye; 4 Department of Neurosurgery, School of Medicine, Istinye University, Goztepe Medical Park Hospital, Istanbul, Türkiye; 5 Neuroscience Research Center, Istinye University, İstanbul, Türkiye

**Keywords:** combined therapy, drug resistance, glioblastoma, selinexor, temozolomide, XPO1

## Abstract

**Introduction:**

Glioblastoma remains one of the most treatment-resistant cancers, and temozolomide (TMZ) resistance is a central barrier to durable clinical response. Inhibition of exportin-1 (XPO1) by selinexor has emerged as a promising strategy to overcome resistance in solid tumors; however, its ability to sensitize TMZ-resistant GBM cells and the underlying mechanisms remain poorly understood.

**Methods:**

We evaluated selinexor and TMZ, alone and in combination, in three GBM cell lines with distinct TMZ sensitivity profiles (LN18, resistant; U87MG, moderate; LN229, sensitive), assessing viability, apoptosis, cell cycle distribution, MMR/survival gene expression, apoptotic protein levels, and long-term colony-forming capacity.

**Results:**

Selinexor potently sensitized GBM cells to TMZ across all three lines, with the most clinically meaningful effects observed in the TMZ-resistant background. The combination produced synergistic growth inhibition and robust apoptotic induction that neither agent achieved independently, driven by convergent suppression of MCL1 and BIRC6, partial restoration of mismatch repair gene expression, and CHK1-mediated checkpoint activation. Combination treatment induced nuclear p53 accumulation, markedly reduced BCL-2, and increased PARP cleavage, and durably suppressed long-term proliferative capacity.

**Discussion:**

These findings position selinexor as a mechanistically rational TMZ sensitizer in resistant GBM and support further preclinical and translational investigation of this combination strategy.

## Graphical Abstract

**Figure:**
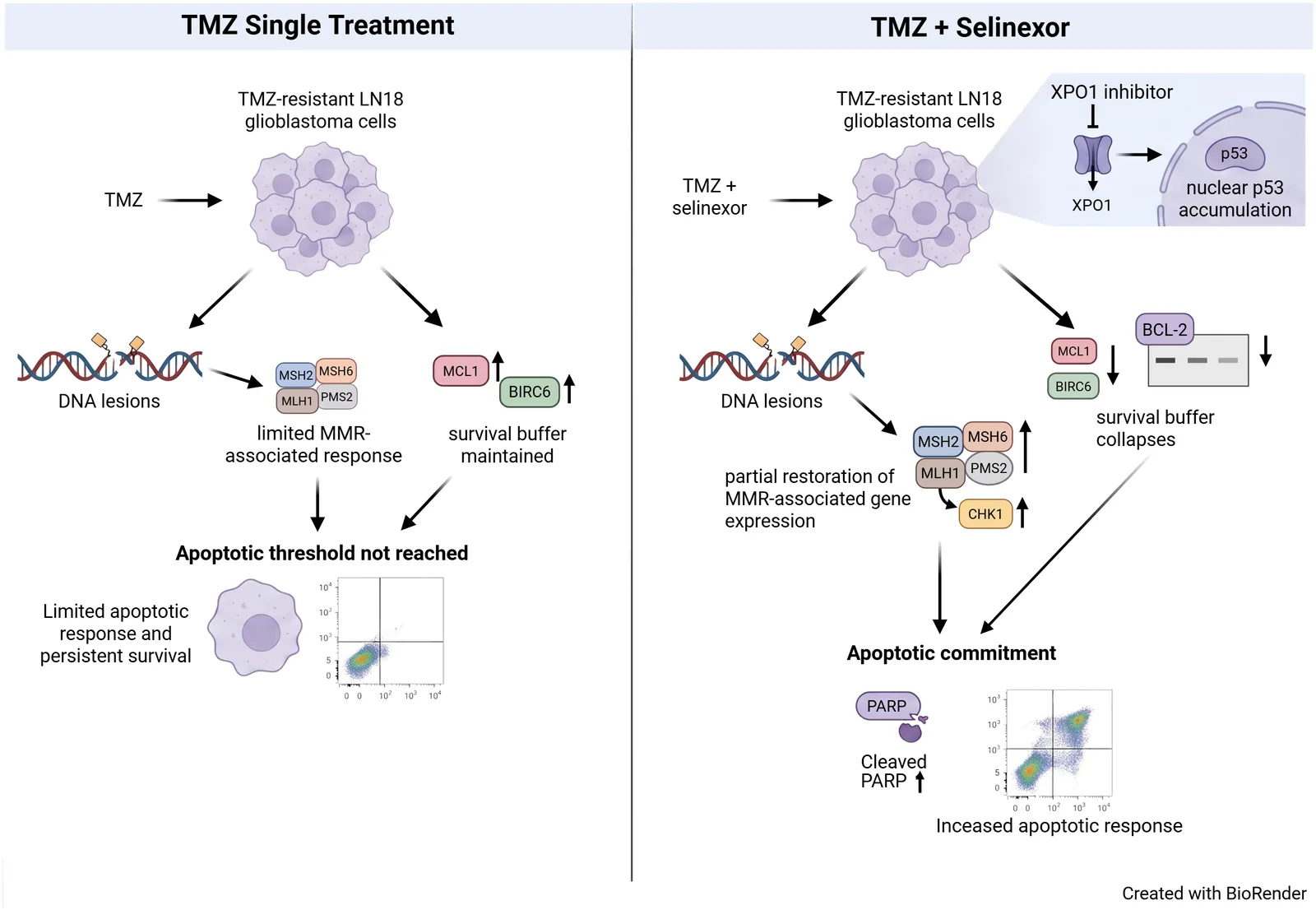

## 1 Introduction

Central nervous system tumors are the most common cancers in children and adolescents (1). Treatment options, including surgery, radiation, and chemotherapy, are tailored based on the tumor type, location, and patient health (2). Glioblastoma, IDH-wildtype (WHO grade 4), classified under the 2021 WHO Classification of CNS Tumors, is the most aggressive subtype of glioma (3).

TMZ (Temozolomide) is an alkylating agent commonly used in glioblastoma chemotherapy that belongs to the imidazotetrazine class (4). TMZ exerts its cytotoxic effects by methylating DNA at the O6 position of guanine residues, forming O6-methylguanine adducts that, when left unrepaired, trigger a futile cycle of mismatch repair, ultimately leading to DNA double-strand breaks and cell death (5). Despite its clinical efficacy, a major limitation of TMZ-based therapy is the development of resistance, which significantly compromises treatment outcomes and contributes to the frequent recurrence observed in patients with GBM after treatment completion.

The MGMT enzyme plays a crucial role in conferring resistance to alkylating agents, such as TMZ (6). The methylation status of the MGMT promoter serves as a significant prognostic marker in gliomas, impacting treatment response and patient survival (7). Although about 50% of GBMs contain a methylated MGMT promoter, resistance to TMZ still persists (8). Acquired resistance to TMZ is associated with alterations in DNA repair pathway components. Proteomic analyses of TMZ-treated GBM cells have revealed broad perturbation of nuclear DNA repair machinery, including MMR components (MSH6 and MSH2), homologous recombination factors and(RAD50, RPA1), ubiquitin-proteasome regulators as well and nucleotide excision repair components (DDB1/2), suggesting that TMZ treatment broadly perturbs the nuclear DNA repair machinery in glioblastoma cells (9). Dysfunction of the mismatch repair (MMR) system is another significant factor, with mutations in MMR genes such as PMS2, MLH1, MSH2, MSH3, and MSH6 contributing to TMZ resistance (10, 11).

Base excision repair (BER) is another resistance mechanism that removes TMZ-induced alkyl adducts before they exert cytotoxic effects (12). Overexpression of BER components, including DNA glycosylase and PARP1, has been observed in gliomas (13, 14), and PARP inhibition has been shown to restore TMZ sensitivity independently of MGMT status (15). Therefore, overcoming temozolomide resistance remains a significant obstacle in the treatment of glioblastoma, highlighting the urgent need for novel therapeutic strategies targeting multiple mechanisms of drug resistance.

Eukaryotic cells rely on nuclear pore complexes (NPCs) for the exchange of large molecules, with XPO1 (CRM1) being the key transporter responsible for exporting a variety of molecules including tumor-suppressor proteins such as p53, RB1, and BRCA1/2, as well as oncogenic regulators including cyclins and TERT, via recognition of Nuclear Export Signal (NES) motifs (16–18). Increased XPO1 expression and activating mutations have been identified across multiple cancer types including solid tumors and hematopoietic malignancies (19–21). Notably, XPO1 overexpression is associated with drug resistance in cancer treatment (21–25). In glioma, XPO1 has been associated with genomic instability, dysregulated biological processes, poor survival, and suppression of glioma cell proliferation upon its inhibition (26, 27).

Cancer cells, with their heightened proliferation rates and metabolism, exhibit substantially greater dependence on nuclear export activity than normal cells, rendering them disproportionately vulnerable to XPO1 inhibition, whereas normal cells remain relatively tolerant of reduced export capacity (28). For this reason, targeting XPO1 emerged as a promising strategy in cancer therapy, leading to the development of SINE compounds, including selinexor (KPT-330), eltanexor (KPT-8602), felezonexor (SL-801), and verdinexor (KPT-335) (20, 29). SINE compounds covalently target Cys528 in the cargo-binding pocket of XPO1, forming a slowly reversible bond that results in low toxicity (30). Preclinical studies in hematologic and solid tumor models have demonstrated growth inhibition, cell cycle arrest, and apoptosis (31–33), with selinexor receiving FDA approval for the treatment of multiple myeloma and diffuse large B cell lymphoma (34, 35).

Although selinexor is primarily used for multiple myeloma, both preclinical and clinical studies have revealed its potential for treating various hematologic and non-hematologic malignancies (36, 37). In glioma, selinexor has been shown to enhance sensitivity to other therapeutic agents (38, 39), with dual XPO1 and Bcl-2/Bcl-xL inhibition showing promising results in GBM model systems (40), and selinexor has further been shown to enhance radiosensitivity in glioblastoma cells both *in vitro* and *in vivo* (41). Most recently, Mapunda et al. (2025) demonstrated that selinexor induces MGMT expression via PKA-CREB signaling in IDH wildtype GBM, identifying MGMT methylation status as a potential determinant of selinexor-TMZ combination sensitivity (42). However, the broader mechanisms by which selinexor sensitizes TMZ-resistant GBM cells across distinct resistance profiles, including its effects on apoptotic signaling, cell cycle regulation, and mismatch repair pathway engagement, remain to be fully characterized. In the present study, we investigated the combined effects of selinexor and TMZ in three GBM cell lines stratified by intrinsic TMZ sensitivity: LN18 (TMZ-resistant), U87MG (TMZ-moderate), and LN229 (TMZ-sensitive). Our aim was to elucidate the molecular mechanisms underlying the differential combinatorial response and identify potential determinants of therapeutic efficacy in a TMZ-resistant setting.

## 2 Materials and methods

### 2.1 Cell lines and monolayer cell culture

Human glioblastoma cell lines LN18 (ATCC, CRL-2610), U87MG (ATCC, HTB-14), and LN229 (ATCC, CRL-2611) were grown as monolayers in 25T flasks in Dulbecco’s modified Eagle’s medium (DMEM; GIBCO) with high glucose content, complete with 10% Fetal Bovine Serum (FBS) and 1% antibiotic/antimycotic. The cells were maintained in 5% CO_2_ at 37 °C.

### 2.2 MTT cell viability assay

To assess the individual and combined effects of selinexor and temozolomide on GBM cell viability, three cell lines were selected based on their TMZ resistance profiles: LN18 (TMZ-resistant), U87MG (TMZ-moderate), and LN229 (TMZ-sensitive). Cells were seeded at 10^4^ cells/well in triplicate on 96-well plates and allowed to adhere for 24 h at 37 °C in a humidified incubator with 5% CO_2_. Following pre-incubation, the cells were treated with selinexor at concentrations ranging from 1 nM to 100 µM or temozolomide at concentrations ranging from 100 nM to 3000 µM. Untreated and 0.1% DMSO vehicle control wells were included in triplicates in each experiment to account for potential solvent effects on cell viability. After 24, 48, and 72 h of incubation, 10 µL of MTT solution (5 mg/mL in PBS) was added to each well and incubated for 1–3 h at 37 °C in a humidified incubator with 5% CO_2_ until formazan crystals were formed. Subsequently, 100 µL of SDS solubilization buffer (0.1 M HCl, 10% SDS) was added to dissolve the formazan crystals, and the plates were incubated at 37 °C for 15 min. Absorbance was measured at 570 nm using a multimode microplate reader (Varioskan LUX; Thermo Fisher Scientific). IC_50_ values were determined using GraphPad Prism (version 8.0.2) by nonlinear regression analysis of dose–response curves, with cell viability (%) plotted against inhibitor concentration and IC_50_ values interpolated from the fitted curves. Data are presented as mean ± SD of the triplicate measurements. The results shown are representative of at least two independent experiments that yielded consistent findings.

### 2.3 Drug combination and synergy analysis

Drug–drug interactions between selinexor and temozolomide were evaluated using SynergyFinder+ (version 3). Raw percentage cell viability data from 48-hour combination treatment experiments were uploaded and analyzed using multiple reference models, including Bliss, Loewe, HSA, and ZIP. ZIP (Zero Interaction Potency) synergy scores are presented as the primary synergy metric, as the ZIP model accounts for the individual dose–response relationships of each agent and calculates synergy as the deviation from expected non-interacting behavior. This approach is particularly appropriate when comparing cell lines with divergent single-agent sensitivities, as it normalizes the combination effect against the inherent potency of each drug rather than against a fixed reference point, thereby enabling a meaningful cross-line comparison of drug interaction patterns. Mean ZIP synergy scores and associated p-values were calculated using SynergyFinder+ and are reported for each cell line.

### 2.4 Apoptosis assay

Apoptosis was assessed using an APC Annexin V/PI staining kit (#640932, BioLegend, USA) according to the manufacturer’s protocol. The culture medium was collected to retain detached apoptotic cells, and adherent cells were detached using trypsin-EDTA and transferred into the same 15 mL Falcon tube. Cells were centrifuged at 210 × g for 5 min, the supernatant was discarded, and the pellet was resuspended in PBS containing 2% FBS and 0.05% sodium azide. Cells were washed twice by centrifugation at 350 × g for 5 min each wash. Following the final wash, the pellet was resuspended in 100 µL of Annexin V binding buffer provided with the kit. Cells were incubated in the dark with 5 µL APC Annexin V and PI (final concentration 1 µg/mL) for 15 min at room temperature. Subsequently, 400 µL of binding buffer was added to each tube, and samples were acquired using a NovoCyte 3005 flow cytometer (Agilent, USA). A minimum of 10,000 events was recorded per sample. The percentage of Annexin V-positive cells was quantified, and the treatment groups were compared with the untreated controls. Data are presented as mean ± SD of triplicate measurements from representative experiments. Statistical analysis was performed using one-way ANOVA, followed by Holm–Šídák or Tukey’s multiple comparison tests, or paired t-test where indicated. To account for inter-experimental variability in baseline apoptosis across independent replicates, a delta apoptosis normalization approach was applied for the stacked bar chart representation. For each combination treatment group, the delta apoptosis attributable to TMZ (Δ TMZ) was calculated by subtracting the mean apoptosis of the untreated control from the mean apoptosis of the TMZ monotherapy group at the corresponding dose. Similarly, the delta apoptosis attributable to selinexor (Δ Sel) was calculated by subtracting the mean apoptosis of the untreated control from the mean apoptosis of the selinexor monotherapy group at the corresponding concentration. These values were then stacked to represent the additive single-agent contribution within each combination condition, allowing direct visual comparison of the individual drug contributions to the overall apoptotic response across treatment groups and cell lines.

### 2.5 Cell cycle assay

Cell cycle distribution was analyzed by propidium iodide (PI) staining (#A2261.0025, PanReac Quimica S. L. U., Spain). Cells were detached using trypsin-EDTA, collected into 15 mL Falcon tubes, and centrifuged at 210 × g for 5 min. The supernatant was discarded, and the pellet was resuspended in Hanks’ Balanced Salt Solution (HBSS) containing 2 mM EDTA and 2% FBS, followed by centrifugation at 250 × g for 5 min. After discarding the supernatant, the cells were fixed in ice-cold 70% ethanol and incubated on ice for 30 min. Following fixation, the cells were washed twice with PBS containing 2% FBS and centrifuged at 250 × g for 5 min per wash. Cells were then treated with RNase A (200 µg/mL; #740505.50, Macherey-Nagel GmbH & Co., Germany) at room temperature for 30 min to eliminate RNA interference with PI staining. Finally, the cells were stained with PI (25 µg/mL) and incubated in the dark at room temperature for 15 min. Cell cycle distribution was acquired using a NovoCyte 3005 flow cytometer (Agilent, USA), and the percentage of cells in the G1, S, and G2/M phases was determined using NovoExpress software. A minimum of 10,000 events was recorded per sample. Cell cycle phase distribution is presented as the percentage of total cells in each phase. The treatment groups were compared descriptively with the untreated controls, with individual data points shown to demonstrate consistency across measurements.

### 2.6 RNA isolation and gene expression analysis

To assess changes in gene expression following treatment, cells were lysed directly on the culture surface using TRIzol reagent and collected into 15 mL Falcon tubes. Cells were centrifuged at 210 × g for 5 min, the supernatant was discarded, and total RNA was isolated according to the TRIzol manufacturer’s protocol. RNA concentrations and purity were determined using a Nabi UV/Vis Nano Spectrophotometer (MicroDigital Co., South Korea). A total of 1000 ng RNA per sample was reverse transcribed into cDNA using a cDNA synthesis kit (#C03-01-20, A.B.T Laboratory Industry, Turkey) according to the manufacturer’s instructions, with thermal cycling performed using a Bio-Rad T100 Thermal Cycler (#1861096, Bio-Rad, Hercules, CA, USA). Quantitative PCR was performed using qPCRBIO SyGreen Blue Mix (#PB20.17-01, PCR Biosystems Inc., USA) according to the manufacturer’s recommendations on a Bio-Rad CFX96 Touch Real-Time PCR Detection System (#1855196, Bio-Rad, USA). Relative gene expression was calculated using the 2^-ΔΔCt method, with GAPDH as the reference gene for normalization. All expression values are presented as fold changes relative to the untreated controls. Data are presented as mean ± SD of the triplicate measurements. The results shown are representative of two independent experiments that yielded consistent findings.

### 2.7 Colony formation assay

To assess the long-term proliferative capacity following combination therapy, a colony formation assay was performed using LN18 cells. Cells were detached using trypsin-EDTA, collected into 15 mL Falcon tubes, and centrifuged at 210 × g for 5 min. The supernatant was discarded, and the pellet was resuspended in fresh complete medium. Cells were counted using a hemocytometer, and 500 cells were seeded per well in 6-well plates. After 24 h of adherence in a humidified incubator at 37 °C with 5% CO_2_, the cells were treated with the indicated concentrations of selinexor and temozolomide, alongside single-agent and untreated controls. After 72 h of treatment, the medium was replaced with fresh complete medium, and the cells were maintained for 10–12 days to allow colony formation. On days 10–12, the medium was removed, and the cells were washed twice with PBS. Cells were fixed with ice-cold methanol for 5 min, followed by two washes with PBS. A 0.5% crystal violet solution (Sigma-Aldrich, #V5265-250mL, Germany) was diluted to 0.25% with PBS and applied to each well for 30 min at room temperature. Crystal violet was then removed, and the wells were rinsed with tap water and allowed to air dry. The wells were scanned, and the colony area was quantified using ImageJ software. The results are presented from a single independent experiment.

### 2.8 Western blot analysis

LN18 glioblastoma cells were treated with Selinexor (200 nM), TMZ (400 µM), the combination, or left untreated for 48 hours. Total protein was extracted using RIPA lysis buffer supplemented with protease inhibitor cocktail. Protein concentration was determined using the Bradford assay. Equal amounts of protein (50 µg) were separated by SDS-PAGE and transferred onto PVDF membranes. Membranes were blocked with 5% non-fat dry milk or 5% BSA in TBST and incubated overnight at 4 °C with primary antibodies against Caspase-9 (#9502, Cell Signaling Technology, 1:1000), PARP (#9542, Cell Signaling Technology, 1:1000), Bcl-2 (551097, BD Biosciences, 1:1000), GAPDH (NA001911200, Nepenthe, 1:5000), and β-actin (#3700, Cell Signaling Technology, 1:2000). After incubation with HRP-conjugated secondary anti-rabbit (#7074, Cell Signaling Technology, 1:2000) or anti-mouse (NA002011100, Nepenthe, 1:5000) IgG antibodies. Protein bands were detected using SuperSignal West Pico PLUS Chemiluminescent Substrate (Thermo Fisher Scientific) and imaged with a ChemiDoc imaging system (Bio-Rad). Band intensities were quantified using ImageJ software (NIH), and target protein levels were normalized to GAPDH and β-actin.

### 2.9 Immunocytochemistry

LN18 cells were seeded onto coverslips in 35-mm dishes (150,000 cells/dish; two replicate dishes per treatment group) and allowed to attach overnight. Cells were treated with Selinexor (200 nM), TMZ (400 µM), the combination, or left untreated for 24 h. Following treatment, cells were fixed with ice-cold methanol, blocked with 1.5% BSA in PBS, and incubated overnight at 4 °C with an anti-p53 primary antibody (sc-98, Santa Cruz Biotechnology; 1:100). Immunostaining was performed using the SensiTek HRP (Anti-Polyvalent) detection kit (ScyTek Laboratories) according to the manufacturer’s instructions. Immunoreactivity was visualized with AEC chromogen (ACK125, ScyTek Laboratories) and slides were counterstained with hematoxylin (BS-005, BestLab) before mounting.

### 2.10 Statistical analysis

Statistical analyses were performed using GraphPad Prism (version 8.0.2). Data are presented as mean ± standard deviation (SD). For comparisons involving more than two groups, one-way analysis of variance (ANOVA) was used, followed by either the Holm–Šídák or Tukey’s multiple comparisons test. For experiments involving matched measurements across independent experimental repeats, a repeated-measures one-way ANOVA or paired t-test was applied. Statistical significance was set at p < 0.05.

## 3 Results

### 3.1 Selinexor sensitized glioblastoma cells to TMZ

To investigate the differential responses of GBM cell lines to selinexor and temozolomide, cell viability was assessed across a range of concentrations and time points. selinexor treatment resulted in a marked, dose-dependent reduction in cell viability, effective at nanomolar concentrations across all cell lines, with the strongest inhibitory effects observed 72 h after treatment. IC_50_ values for selinexor were 0.286 µM in LN18, 0.072 µM in U87MG, and 0.288 µM in LN229 cells (**Figures 1A–C**, upper panels). The temozolomide IC_50_ values were consistent with the known TMZ resistance profiles of each cell line, with LN18 showing the highest IC_50_ (1729 µM), U87MG displaying intermediate sensitivity (698 µM), and LN229 being the most sensitive (228.5 µM) at 48 h post-treatment (**Figures 1A–C**, lower panels).

**Figure 1:**
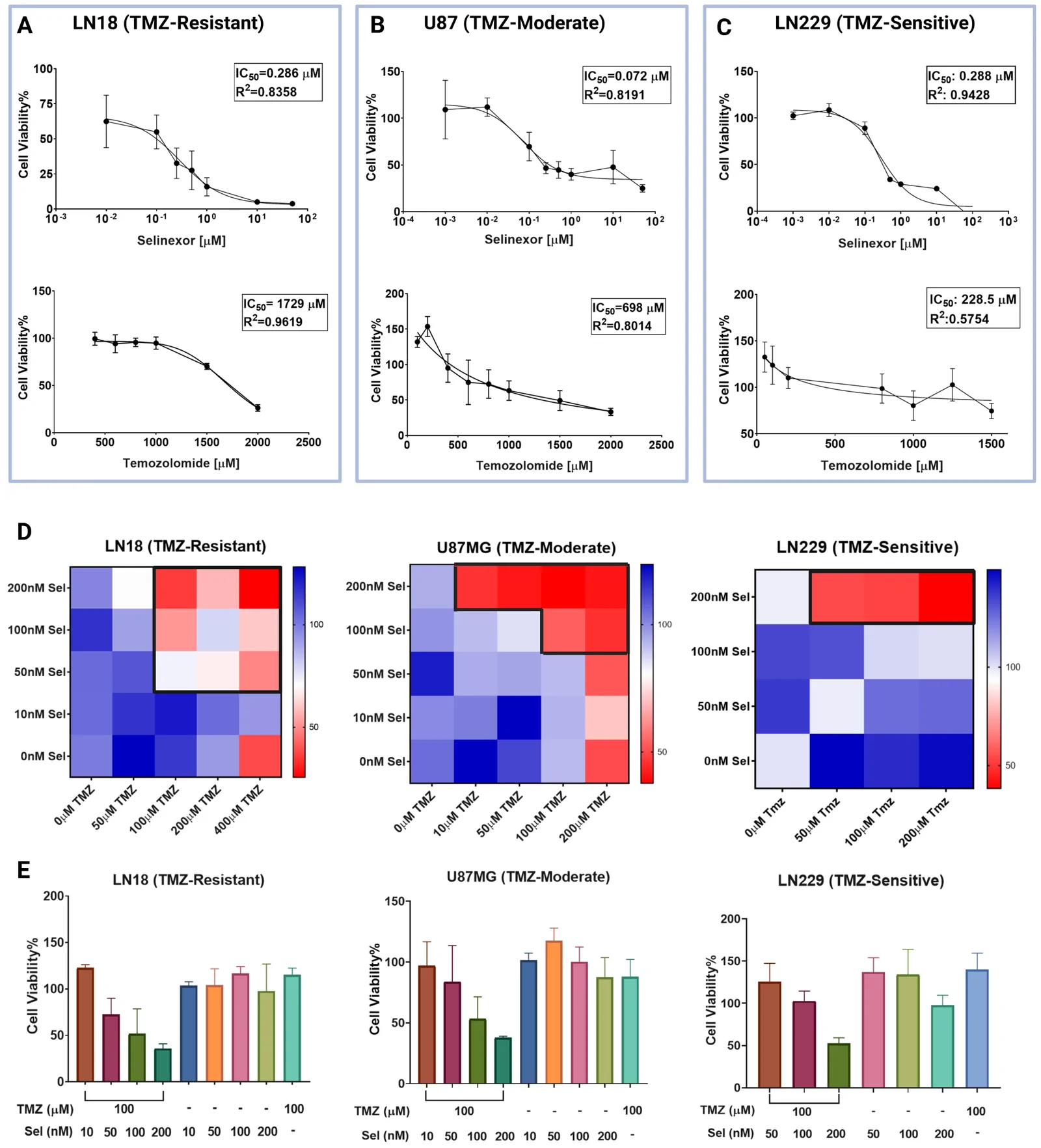
Selinexor sensitized GBM cell lines to temozolomide in a concentration-dependent manner. **(A-C)** Dose-response curves for selinexor (upper panels) and temozolomide (lower panels) in LN18 (TMZ-resistant), U87MG (TMZ-moderate), and LN229 (TMZ-sensitive) GBM cell lines, respectively. Cell viability was assessed using the MTT assay 48 h after treatment with increasing concentrations of each agent. IC_50_ values and R² were determined using nonlinear regression curve fitting. Data are presented as mean ± SD of three independent experiments. **(D)** Heatmap representation of cell viability (%) following a 48-hour treatment with a dose matrix of selinexor and temozolomide across all three cell lines. The color scale represents the percentage of cell viability relative to the untreated controls; blue indicates growth inhibition, and red indicates values exceeding the untreated baseline. Black borders indicate the dose combinations selected for subsequent mechanistic analyses based on consistent and biologically relevant growth inhibition. **(E)** Cell viability following treatment with escalating selinexor concentrations in the presence of 100 µM temozolomide (bracketed bars) or selinexor alone, at 48 h, across LN18, U87MG, and LN229 cell lines. Data are presented as mean ± SD of the triplicate measurements.

A 4×4 selinexor–TMZ concentration matrix was employed as an exploratory screening approach to identify dose ranges that produce biologically relevant combinatorial effects and guide the selection of conditions for subsequent mechanistic analyses at 48 h. Heatmap analysis revealed a clear concentration-dependent modulation of cell growth in response to the combination treatment across all cell lines (**Figure 1D**). While low-dose combinations showed limited effects, intermediate to high concentrations of selinexor markedly enhanced the response to TMZ, particularly at moderate doses. Notably, specific dose regions exhibited a stronger growth-inhibitory effect than either agent alone (black borders in **Figure 1D**), indicating that selinexor increases cellular sensitivity to TMZ in a concentration-dependent manner. The magnitude and pattern of the combinatorial response varied between cell lines owing to their distinct TMZ sensitivity profiles.

To further characterize the sensitizing effect of selinexor, TMZ was kept constant at 100 µM, while selinexor concentrations were systematically increased across all three cell lines at 48 h (**Figure 1E**). Increasing selinexor doses resulted in a progressive reduction in cell viability in the presence of fixed TMZ, a pattern not observed when the same selinexor concentrations were administered alone, suggesting a combination-specific enhancement of growth inhibition. In LN18 cells, increasing selinexor concentration from 10 to 200 nM led to an approximately 3–4-fold decrease in viability compared to the lowest selinexor dose. A similar dose-dependent potentiation was observed in U87MG cells, resulting in an approximate 2–3-fold reduction in cell viability. In LN229 cells, which are intrinsically more sensitive to TMZ, selinexor co-treatment also reduced viability in a concentration-dependent manner, with the strongest inhibitory effects observed at higher selinexor doses. Given the large number of tested combinations, detailed statistical analyses are presented in **Supplementary Figure 1**, focusing on combinations that reached significance and demonstrating that a substantial subset of selinexor–TMZ combinations resulted in significantly greater growth inhibition than monotherapies, with the timing and magnitude of significance varying across cell lines. Based on these data, selinexor concentrations of 50–200 nM combined with 100–200 µM TMZ were selected as the most effective and consistent dose range for subsequent analyses, with an additional 400 µM TMZ included for LN18 cells to capture the full range of selinexor-mediated modulation.

Together, these findings indicate that selinexor enhances TMZ-mediated growth inhibition in a dose-dependent manner across GBM cell lines, with the most pronounced effect observed in the TMZ-resistant LN18 cells. These results prompted further investigation into the mechanistic basis of sensitization.

To quantitatively assess drug–drug interactions in GBM cells, synergy analysis was performed using the Zero Interaction Potency (ZIP) model implemented in SynergyFinder+. ZIP synergy maps generated from 48-hour viability matrices revealed positive drug interactions between selinexor and TMZ across all three GBM cell lines, with distinct patterns reflecting the TMZ sensitivity profile (**Figure 2**).

**Figure 2:**
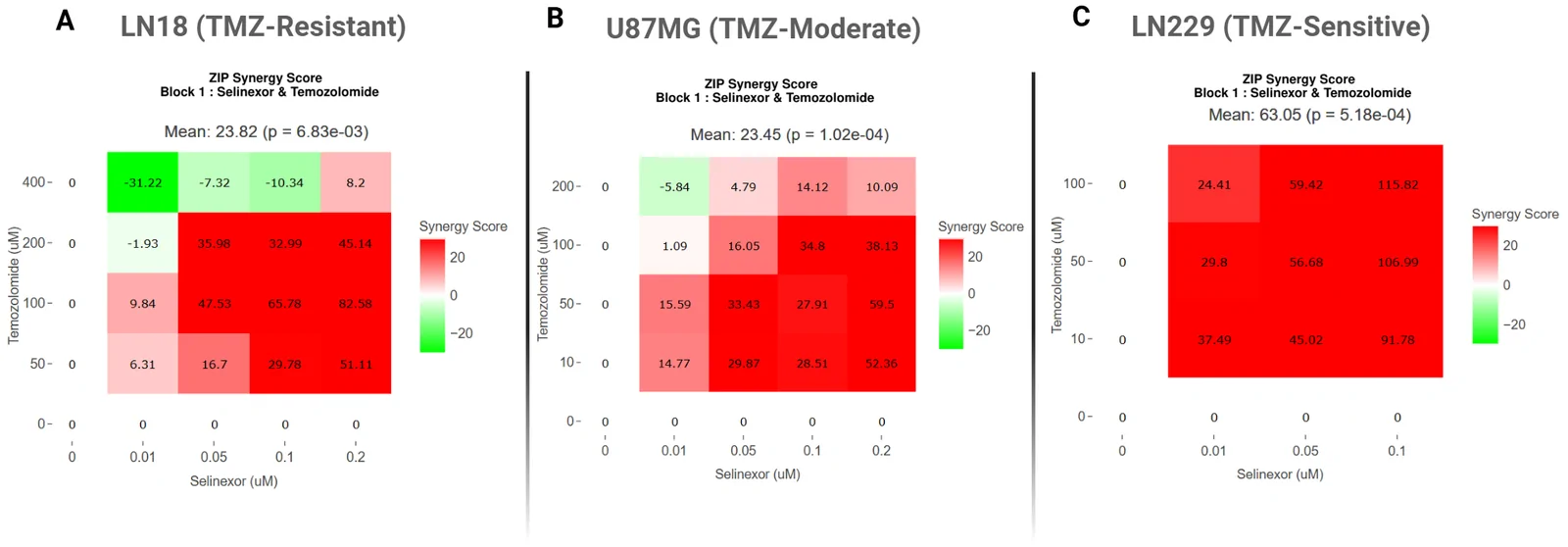
Selinexor and temozolomide exhibited synergistic interactions across GBM cell lines, with distinct dose-dependent patterns reflecting TMZ sensitivity profiles. **(A-C)** ZIP synergy score heatmaps generated from 48-hour cell viability dose–response matrices for LN18 (TMZ-resistant), U87MG (TMZ-moderate), and LN229 (TMZ-sensitive) GBM cell lines. The color scale represents ZIP synergy scores; red indicates synergistic interactions, and green indicates antagonism. The mean ZIP synergy scores and associated p-values are displayed above each heatmap. Synergy analysis was performed using SynergyFinder+ (version 3). selinexor concentrations are shown on the x-axis (µM), and temozolomide concentrations are shown on the y-axis (µM).

In TMZ-resistant LN18 cells, meaningful ZIP synergy scores were observed specifically at selinexor concentrations of 0.05–0.2 µM combined with 50–200 µM TMZ, with a mean ZIP score of 23.82 (p=6.83×10^-^³), whereas low selinexor doses and the highest TMZ concentration (400 µM) showed limited or negative interaction scores (**Figure 2A**). This dose-dependent synergistic pattern in a TMZ-resistant background is particularly noteworthy, as TMZ alone exerts minimal cytotoxic effects in LN18 cells, indicating that the observed synergy reflects a genuine selinexor-mediated sensitization to TMZ rather than simple additivity.

In U87MG cells, positive ZIP synergy was detected across a broader dose range, with the highest scores at selinexor concentrations ≥0.05 µM combined with 10–100 µM TMZ, yielding a mean ZIP score of 23.45 (p=1.02×10^-4^), consistent with intermediate TMZ sensitivity (**Figure 2B**).

In LN229 cells, uniformly high ZIP synergy scores were observed across nearly all tested combinations, with the highest mean ZIP score of 63.05 (p=5.18×10^-4^) (**Figure 2C**). However, this pattern should be interpreted in the context of LN229’s intrinsic TMZ sensitivity; both agents are individually active in this line, and the high ZIP scores reflect consistent additive-to-synergistic interactions rather than resistance-overcoming sensitization. The clinically relevant finding remains the dose-dependent synergy achieved in TMZ-resistant LN18 cells, where selinexor converts a TMZ-insensitive line into one that responds meaningfully to combination treatment.

Together, these synergy data, integrated with the viability heatmap and dose-response findings, informed the selection of biologically relevant dose combinations for subsequent mechanistic analyses.

### 3.2 Selinexor and TMZ combination overcomes apoptotic resistance in TMZ-resistant GBM cells

To evaluate whether selinexor enhances TMZ-induced apoptosis beyond the single-agent effects, Annexin V/PI staining was performed across all three GBM cell lines following treatment with a 3×3 dose matrix of selinexor and TMZ alongside single-agent and untreated controls. Representative flow cytometry dot plots for LN18 cells demonstrated a visually apparent increase in the apoptotic population in combination-treated cells compared to either single-agent or untreated controls (**Figure 3A**).

**Figure 3:**
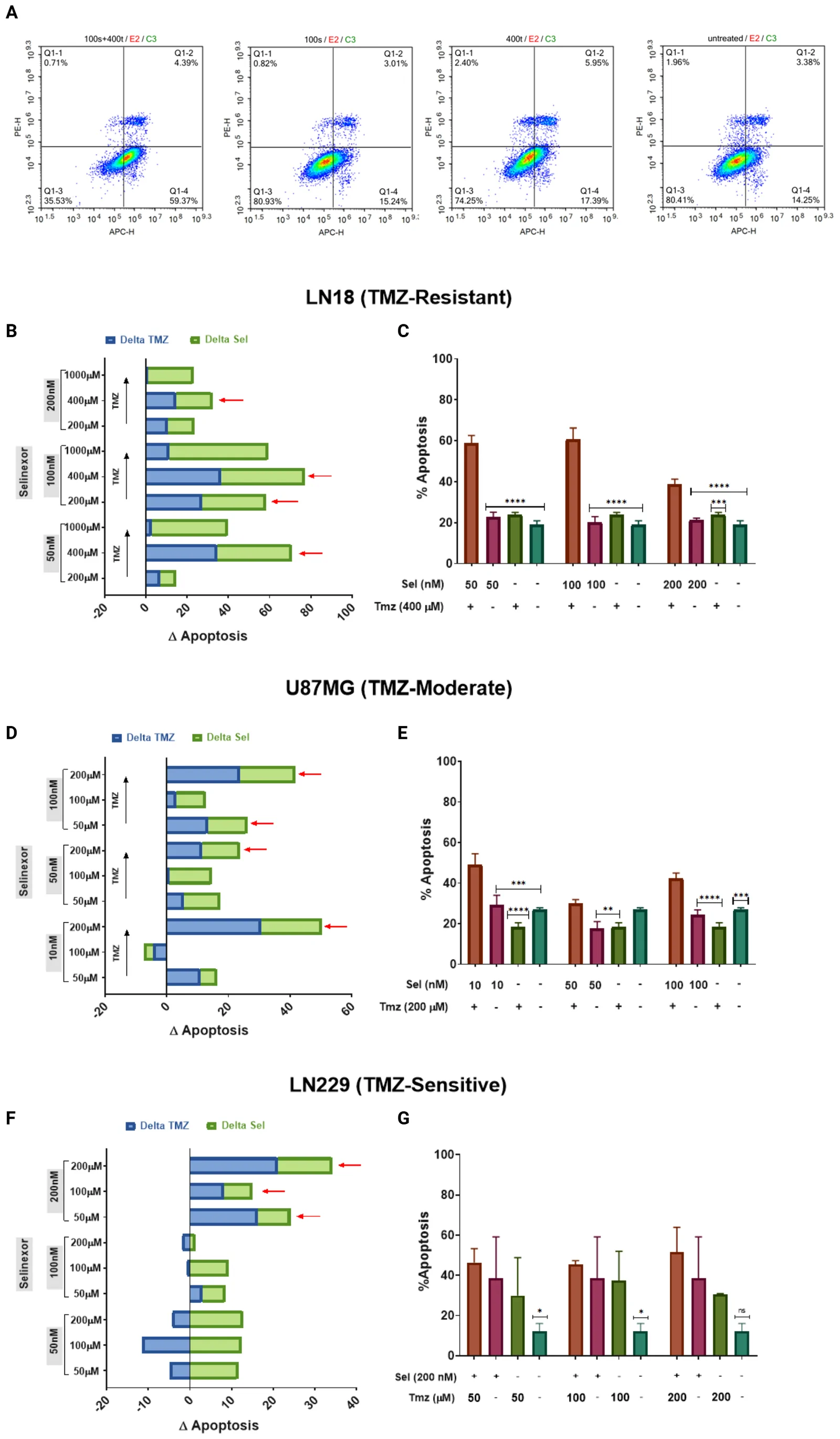
Combination of Selinexor and TMZ overcame apoptotic resistance in TMZ-resistant GBM cells. **(A)** Representative flow cytometry dot plots showing Annexin V/PI staining in LN18 cells following treatment with 100 nM selinexor + 400 µM TMZ (combination), 100 nM selinexor alone, 400 µM TMZ alone, and untreated control. Q1-3: live cells (Annexin V^-^/PI^-^); Q1-4: early apoptotic cells (Annexin V^+^/PI^-^); Q1-2: late apoptotic/necrotic cells (Annexin V^+^/PI^+^); Q1-1: necrotic cells (Annexin V^-^/PI^+^). The percentages of cells in each quadrant are indicated. **(B, D, F)** Delta apoptosis horizontal bar charts for LN18, U87MG, and LN229 cells, showing the difference in apoptosis between each combination group and the corresponding single agent (delta TMZ, blue; delta Sel, green). Positive values indicated that the combination exceeded the respective single agent. The red arrows indicate combinations with the most balanced and meaningful bidirectional contributions from both agents. **(C, E, G)** Percentage apoptosis bar charts for LN18, U87MG, and LN229 cells, respectively, showing combination treatment compared to single agent and untreated controls at fixed TMZ concentrations (400 µM for LN18, 200 µM for U87MG) across escalating selinexor doses. For LN229 cells, a fixed selinexor concentration (200 nM) with escalating TMZ doses is shown. Data are presented as mean ± SD of triplicate measurements from representative experiments. Statistical significance was determined using one-way ANOVA with Holm–Šídák multiple comparison test **(C)**, one-way ANOVA with Tukey’s multiple comparison test **(E)**, or paired t-test **(G)**. ****p<0.0001, ***p<0.001, **p<0.01, *p<0.05, ns, not significant.

To quantify the individual contribution of each drug to the combination effect, delta apoptosis values were calculated by subtracting single-agent apoptosis values from the corresponding combination values, yielding delta TMZ and delta Sel for each dose pair. Positive delta values indicated that the combination exceeded the effect of the respective single agent. Combinations in which both delta TMZ and delta Sel were simultaneously positive and approximately equal in magnitude were considered to reflect a balanced contribution from both agents, representing the most meaningful combinatorial interactions. Among these, combinations that yielded the highest overall apoptosis values were prioritized. These combinations are indicated by red arrows in the delta apoptosis panel (**Figure 3**).

In LN18 cells, delta apoptosis analysis confirmed that the combination effect exceeded that of both single agents independently across multiple dose pairs, with the most balanced bidirectional contributions observed at intermediate selinexor doses (**Figure 3B**, red arrows). Direct statistical comparison confirmed significantly greater apoptosis induction in the combination groups than in the groups treated with selinexor and TMZ alone across all three selinexor doses tested, with the highest absolute apoptotic yields observed at 400 µM TMZ combinations (**Figure 3C**; mean differences 14.77–41.43%, adjusted p ranging from 0.000005 to 0.000121). Notably, the ZIP synergy analysis identified antagonistic interaction scores at 400 µM TMZ in LN18 cells (**Figure 2A**). However, direct apoptosis comparison demonstrated that combination treatment at this dose significantly exceeded both single agents in terms of absolute apoptotic yield. This apparent discrepancy reflects a known limitation of mathematical synergy models: ZIP scores quantify deviation from expected non-interacting behavior based on fitted dose-response curves and can register antagonism even when the combination remains functionally superior to monotherapy. This phenomenon has been documented in the pharmacological literature and highlights the importance of complementing synergy modeling with direct functional readouts (43, 44). Supporting apoptosis data at 200 µM TMZ, which falls within the synergistic ZIP score region for LN18 cells, are provided in **Supplementary Figure 2** and demonstrate consistent combination superiority at lower TMZ concentrations.

In U87MG cells, delta apoptosis analysis identified several combinations with balanced bidirectional contributions from both agents, with the most pronounced and balanced effects observed at 200 µM TMZ combined with increasing selinexor concentrations, as indicated by the red arrows in **Figure 3D**. Direct statistical comparison confirmed significantly greater apoptosis induction in the combination groups than in the single-agent groups across multiple dose combinations (**Figure 3E**; mean differences 11.60–30.63%, adjusted p ranging from <0.0001 to 0.0022).

In LN229 cells, delta apoptosis values were generally smaller in magnitude than those in LN18 and U87MG cells, consistent with the higher baseline single-agent activity in this TMZ-sensitive line (**Figure 3F**, red arrows). Statistical significance was reached relative to untreated controls but not between the combination and single-agent groups in most dose pairs (**Figure 3G**; combination vs untreated, p = 0.0434 at 50 µM and p = 0.0253 at 100 µM TMZ, and p = 0.1794 at 200 µM TMZ), likely reflecting the high inter-experimental variability in this line and the ceiling effect attributable to intrinsic TMZ sensitivity, where both agents are already individually active.

### 3.3 Selinexor and TMZ combination induces cell line-specific cell cycle redistribution in GBM cells

To investigate the effects of the combination of selinexor and TMZ on cell cycle distribution, propidium iodide (PI) staining was performed across all three GBM cell lines following treatment with the selected dose matrix, alongside single agents and untreated controls. Representative cell cycle histograms for LN229 cells are shown in **Figure 4A**, demonstrating a visually apparent increase in the G2/M population and redistribution away from G1 in combination-treated cells compared to those treated with single agents and the untreated controls.

**Figure 4:**
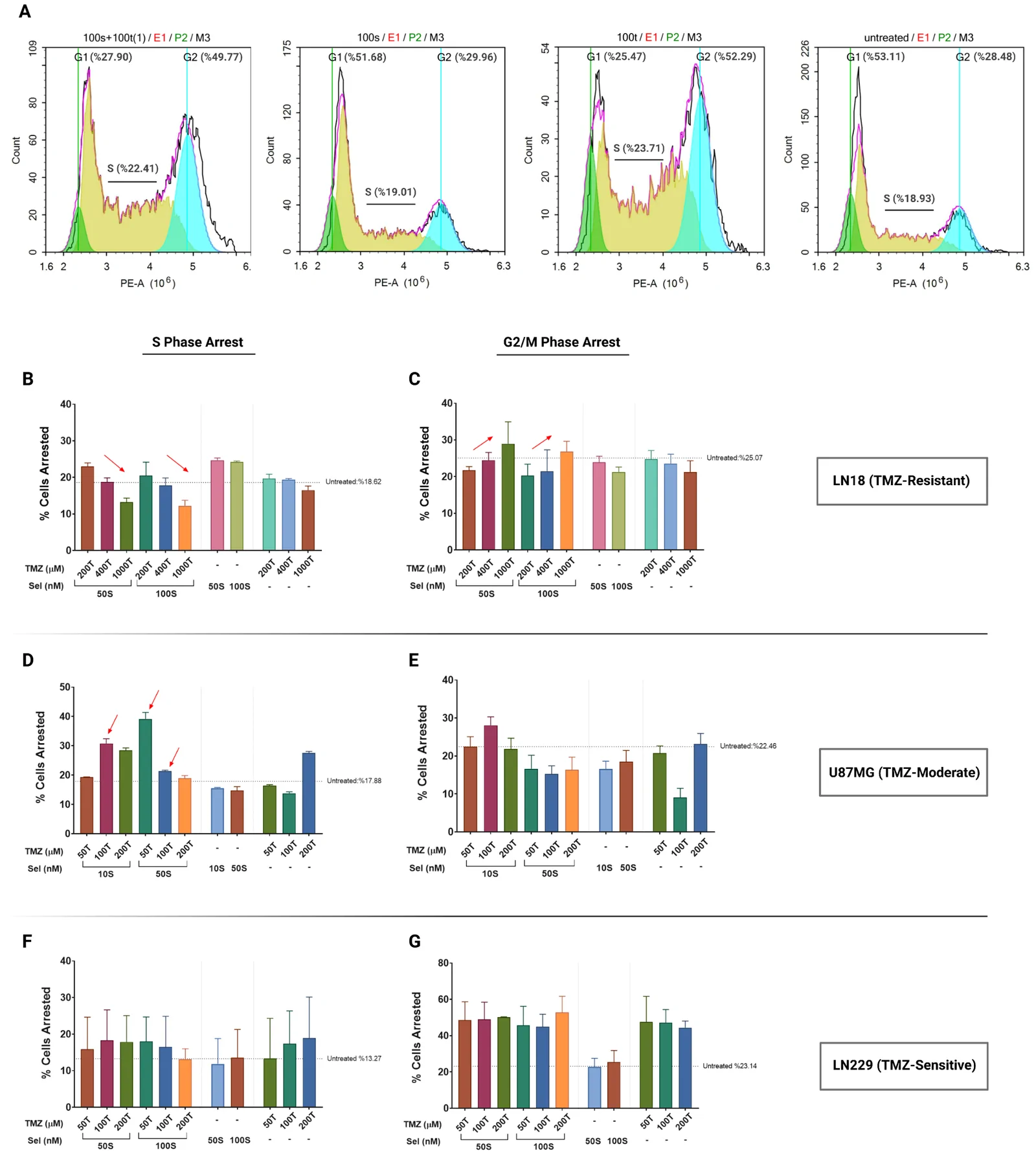
The selinexor and TMZ combination induced cell line-specific cell cycle redistribution in GBM cells. **(A)** Representative PI staining histograms showing cell cycle distribution in LN229 cells following treatment with 100 nM selinexor + 100 µM TMZ (combination), 100 nM selinexor alone, 100 µM TMZ alone, and untreated control. G1, S, and G2/M phases are indicated for each condition. **(B, C)** Percentage of LN18 cells in S phase and G2/M phase, respectively, following treatment with the indicated selinexor and TMZ dose combinations and single agents. The red arrows indicate the combination groups that exhibited the most apparent dose-dependent redistribution pattern. The dashed line indicates the untreated baseline. **(D, E)** Percentage of U87MG cells in the S phase and G2/M phase, respectively. The red arrows indicate the combination groups with the most pronounced S-phase accumulation above the untreated baseline. **(F, G)** Percentage of LN229 cells in the S phase and G2/M phase, respectively. G2/M elevation was observed across all treated groups, consistent with TMZ-driven checkpoint activation in the TMZ-sensitive line. Data are presented as mean ± SD of triplicate measurements from representative experiments. Cell cycle phase distributions were presented descriptively, and no inferential statistical analysis was performed. Dashed lines indicate the untreated baseline values for each cell line. S, Selinexor (nM); T, Temozolomide (µM).

Cell cycle effects were distinct across cell lines, reflecting their differential TMZ sensitivity profiles and were consistent with the apoptosis findings described above.

In LN18 cells, no statistically significant differences in S phase or G2/M phase distribution were observed between the combination and single-agent groups relative to the untreated controls (**Figures 4B, C**). However, a dose-dependent redistribution pattern was observed in the combination groups; as the TMZ concentration increased within fixed selinexor dose blocks, the S phase percentage progressively decreased, whereas the G2/M phase percentage correspondingly increased, as indicated by the red arrows in **Figures 4B, C**. This inverse dose-dependent relationship between the S and G2/M phases was not observed in the single-agent groups, where both phases remained relatively stable across doses. Although these trends did not reach statistical significance, their internal consistency across multiple dose combinations suggests a biologically relevant cell cycle redistribution driven specifically by the combination, rather than by either agent alone.

In U87MG cells, S-phase accumulation was the predominant combination-specific cell cycle effect, with selected combination groups showing markedly elevated S-phase percentages compared to single agents and untreated controls (**Figure 4D**, red arrows). The most pronounced S phase accumulation was observed at 10 nM and 50 nM selinexor combined with higher TMZ concentrations, with combination groups clearly exceeding the untreated baseline of 17.88%. G2/M phase distribution showed no consistent combination-specific pattern in U87MG cells (**Figure 4E**), indicating that S phase accumulation, rather than G2/M arrest, is the predominant cell cycle effect of the combination in this cell line.

In LN229 cells, the G2/M phase was markedly elevated across all TMZ-treated groups compared to the untreated controls and selinexor alone groups (**Figure 4G**). This uniform elevation across treatment conditions, including TMZ alone, is consistent with the intrinsic TMZ sensitivity of LN229 cells and the known ability of TMZ-induced DNA damage to trigger G2/M checkpoint activation, even as a monotherapy in sensitive lines. S phase distribution in LN229 cells showed no consistent combination-specific pattern (**Figure 4F**). The combination did not produce a distinct cell cycle profile beyond what TMZ alone achieved in this sensitive line, further supporting the interpretation that the marginal combinatorial benefit in LN229 cells reflects a ceiling effect, where TMZ is already maximally engaging the DNA damage response machinery.

Collectively, the cell cycle data revealed distinct line-specific responses — a dose-dependent S to G2/M redistribution trend in resistant LN18 cells, S phase accumulation in moderate U87MG cells, and TMZ-driven G2/M elevation in sensitive LN229 cells — that together reflect the differential engagement of DNA damage checkpoints across GBM lines with varying TMZ sensitivity profiles.

### 3.4 Combination treatment modulates key gene networks involved in therapy resistance

To elucidate the molecular mechanisms underlying the observed pro-apoptotic and antiproliferative effects of the combination of selinexor and TMZ, quantitative real-time PCR analysis was performed on key regulatory genes involved in mismatch repair, survival signaling, and DNA damage checkpoint activation. Based on the dose-response and apoptosis data, 100 nM and 200 nM selinexor were selected for gene expression analysis, combined with 400 µM TMZ for LN18 and 100 µM TMZ for LN229, alongside matched single-agent controls. Gene expression was presented as fold-change relative to untreated controls (2^-ΔΔCt^), where values below 1.0 indicate downregulation and values above 1.0 indicate upregulation of gene expression. Due to the inter-experimental variability inherent to the sensitive LN229 cell line and the descriptive nature of these analyses, the results were interpreted as consistent directional trends across independent experiments rather than statistically validated differences. The time points shown for each gene represent the point of maximal combination-specific effect as determined by visual inspection across 24, 48, and 72 h measurements.

#### 3.4.1 Mismatch repair genes

To assess the MMR pathway response to combination therapy, MSH2, MSH6, PMS2, and MLH1 expression was examined in both LN18 and LN229 cells. Cross-line comparison of MMR gene expression revealed a striking contrast that provides mechanistic insight into the differential TMZ sensitivity of these lines.

In LN18 cells, MMR gene expression was generally suppressed at or below the untreated baseline across all treatment groups, consistent with MMR pathway dysregulation as an intrinsic feature of TMZ resistance in this cell line. However, a consistent pattern emerged across all four MMR genes: the combination groups showed higher expression than both single-agent groups at the selected time points, suggesting that the combination partially restores MMR-mediated damage recognition, which neither selinexor nor TMZ achieves independently. MSH2 expression at 72 h was suppressed below that of the untreated group in all groups, yet the combination groups showed approximately 0.80 and 0.65 fold expression for 100 nM and 200 nM selinexor combinations, respectively, clearly exceeding the sel alone values of approximately 0.35 and 0.30 fold (**Figure 5A**). MSH6 at 24 h showed a transient elevation specifically in combination groups, reaching approximately 2.0–2.5 fold above the untreated group, while the single-agent groups remained at or below baseline, representing the most dramatic combination-specific MMR induction observed in LN18 cells (**Figure 5B**). PMS2 at 48 h similarly showed higher expression in the combination groups than in both single-agent groups, despite all values remaining below the untreated baseline (**Figure 5C**). MLH1 at 24 h showed a more variable pattern than the other MMR genes, with inconsistent separation between the combination and single-agent groups. Notably, the 200 nM selinexor combination group showed the highest MLH1 expression among all treated groups, suggesting a dose-dependent contribution of selinexor to MLH1 restoration at this concentration (**Figure 5D**).

**Figure 5:**
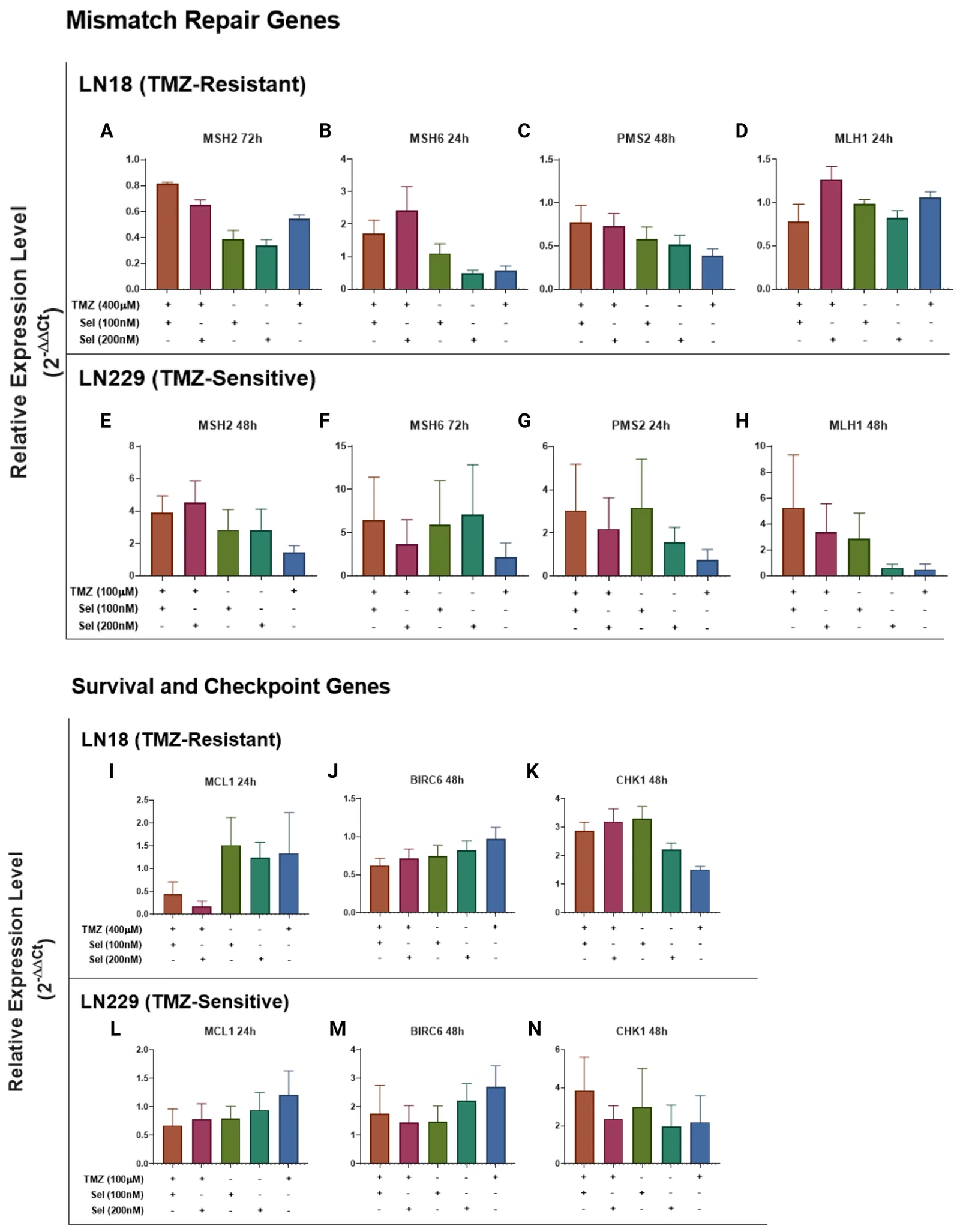
The selinexor and TMZ combination modulated MMR, survival, and checkpoint gene expression in a cell line and time-dependent manner. **(A-D)** Mismatch repair gene expression in LN18 (TMZ-resistant) cells: MSH2 at 72 h **(a)**, MSH6 at 24 h **(b)**, PMS2 at 48 h **(c)**, and MLH1 at 24 h **(d)**. Combination groups consistently showed higher MMR gene expression than single-agent groups, suggesting partial re-engagement of the damage recognition machinery that neither single agent achieves independently. **(E-H)** Mismatch repair gene expression in LN229 (TMZ-sensitive) cells: MSH2 at 48 h **(e)**, MSH6 at 72 h **(f)**, PMS2 at 24 h **(g)**, and MLH1 at 48 h **(h)**. All selinexor-containing groups showed substantially elevated MMR gene expression compared to the untreated controls, consistent with constitutively active and intact MMR engagement in this TMZ-sensitive line. **(I-K)** Survival and checkpoint gene expression in LN18 cells: MCL1 at 24 h **(i)**, BIRC6 at 48 h **(j)**, and CHK1 at 48 h **(k)**. Combination treatment uniquely suppressed MCL1 and BIRC6 below the levels observed with single agents, whereas single agents showed expression at or above the untreated baseline. CHK1 was elevated in combination and selinexor-containing groups above TMZ alone, consistent with checkpoint activation in response to unrepaired DNA damage. **(L-N)** Survival and checkpoint gene expression in LN229 cells: MCL1 at 24 h **(l)**, BIRC6 at 48 h **(m)**, and CHK1 at 48 h **(n)**. MCL1 was modestly suppressed across all groups without compensatory single-agent induction. TMZ alone resulted in the highest BIRC6 expression, which was attenuated by combination therapy. CHK1 was highest in the 100 nM selinexor combination group, consistent with G2/M checkpoint activation observed in LN229 cell cycle analysis. All values are presented as relative expression levels (2^-ΔΔCt^) normalized to untreated controls and the GAPDH reference gene. Data are presented as mean ± SD of triplicate measurements from the representative experiments. The results were interpreted as consistent directional trends across independent experiments. Treatment groups: TMZ, temozolomide; Sel, selinexor. The concentrations are indicated on the x-axis of each panel.

In contrast, LN229 cells demonstrated substantially elevated MMR gene expression across the combination and single-agent groups compared to the untreated controls, indicating constitutively active MMR engagement in this TMZ-sensitive line. MSH2 at 48 h showed the clearest combination-specific elevation, with combination groups reaching approximately 3.8–4.3 fold above the untreated group and exceeding both the Sel alone and TMZ alone groups (**Figure 5E**). For MSH6, PMS2, and MLH1, all treated groups, including those treated with single agents, showed elevated expression compared to the untreated controls, with TMZ alone consistently showing the lowest values among the treated groups (**Figures 5F–H**). This pattern suggests that in LN229 cells, selinexor-containing groups, whether alone or in combination, drive MMR gene expression, while TMZ alone contributes less to MMR induction in this line, despite its high cytotoxic activity.

Together, the MMR gene expression data reveal a fundamental difference between the two lines: LN229 cells constitutively engage the full MMR cascade in response to treatment, while LN18 cells require the combination to partially restore MMR expression, which neither single agent achieves alone, consistent with MMR dysregulation as a defining feature of TMZ resistance in this line.

#### 3.4.2 Survival and checkpoint genes

MCL1, BIRC6, and CHK1 expression levels were examined to assess the impact of combination treatment on survival signaling and checkpoint activation in both cell lines.

In LN18 cells, MCL1 expression at 24 h was markedly suppressed in the combination groups compared to that with single agents, with combination values of approximately 0.35 and 0.10 fold for 100 nM and 200 nM selinexor, respectively, while selinexor alone and TMZ alone groups showed expression at or above the untreated baseline (**Figure 5I**). This pattern, where single agents induce a compensatory MCL1 survival response that the combination uniquely abolishes, represents the most striking combination-specific molecular finding in LN18 cells and provides a direct mechanistic explanation for the superior apoptosis induction observed in combination-treated resistant cells. BIRC6 expression at 48 hours similarly showed the lowest values in combination groups compared to single agents, with TMZ alone showing the highest BIRC6 expression, consistent with a TMZ-induced survival escape response that is prevented by selinexor co-treatment (**Figure 5J**). CHK1 expression at 48 h was elevated in the combination and sel-containing groups compared to that in the TMZ alone group in LN18 cells, with combination values reaching approximately 2.5–3.0 fold above the untreated group (**Figure 5K**). This CHK1 upregulation in the combination groups is consistent with the activation of the G2/M checkpoint in response to unrepaired DNA damage, providing a molecular bridge to the dose-dependent S to G2/M redistribution trend observed in LN18 cell cycle analysis.

In LN229 cells, MCL1 expression at 24 h showed modest suppression in the combination groups relative to the untreated group, with all groups remaining below or near the baseline (**Figure 5L**). Unlike LN18, the single-agent groups did not show compensatory MCL1 induction, consistent with the lower MCL1 survival dependence in this TMZ-sensitive line. BIRC6 at 48 hours showed a pattern similar to LN18 — TMZ alone showed the highest expression while combination groups were lower — though the absolute magnitude of the effect was greater in LN229, where TMZ alone reached approximately 2.7 fold above untreated compared to 1.0 fold in LN18 (**Figure 5M**). This larger TMZ-induced BIRC6 response in LN229 cells likely reflects the higher TMZ activity in this sensitive line, triggering a stronger compensatory survival response, which is attenuated by the combination. CHK1 at 48 h showed elevated expression across all treated groups in LN229, with the 100 nM selinexor combination group reaching the highest values of approximately 3.5–4.0 fold above untreated (**Figure 5N**). Although the TMZ alone and Sel alone groups also showed elevated CHK1 expression, the 100 nM selinexor combination consistently showed the highest expression among the treated groups, suggesting that selinexor co-treatment amplifies CHK1-mediated checkpoint activation beyond that achieved by TMZ alone, even in a sensitive line. This is consistent with the G2/M elevation observed across all treatment groups in LN229 and supports CHK1 as a shared checkpoint mediator in both resistant and sensitive GBM lines, driven more selectively by the combination in LN18 and more broadly across all treatments in LN229.

### 3.5 Selinexor and TMZ combination suppresses long-term proliferative capacity in TMZ resistant cells

To assess the long-term antiproliferative effects of the selinexor and TMZ combination, a colony formation assay was performed using LN18 cells. Representative images and quantified colony area percentages are presented in **Figure 6**. At 100 nM selinexor combined with 400 µM TMZ, the colony area was reduced to approximately 22% compared to 55% for selinexor alone and 24% for TMZ alone, with the untreated baseline reaching approximately 53% (**Figures 6A, C**). The most dramatic suppression was observed with 200 nM selinexor combined with 400 µM TMZ, where the colony area was reduced to approximately 6%, representing an approximately 10-fold reduction compared to selinexor alone at the same dose (58%) and a 4-fold reduction compared to TMZ alone (24%) (**Figures 6B, C**). Critically, neither agent at either dose meaningfully reduced colony formation below untreated levels, confirming the intrinsic TMZ resistance of LN18 cells and the inability of selinexor alone to suppress long-term proliferation at these concentrations. This combination uniquely abolished colony-forming capacity in a dose-dependent manner, with the 200 nM selinexor combination producing near-complete growth suppression that neither drug achieved independently.

**Figure 6:**
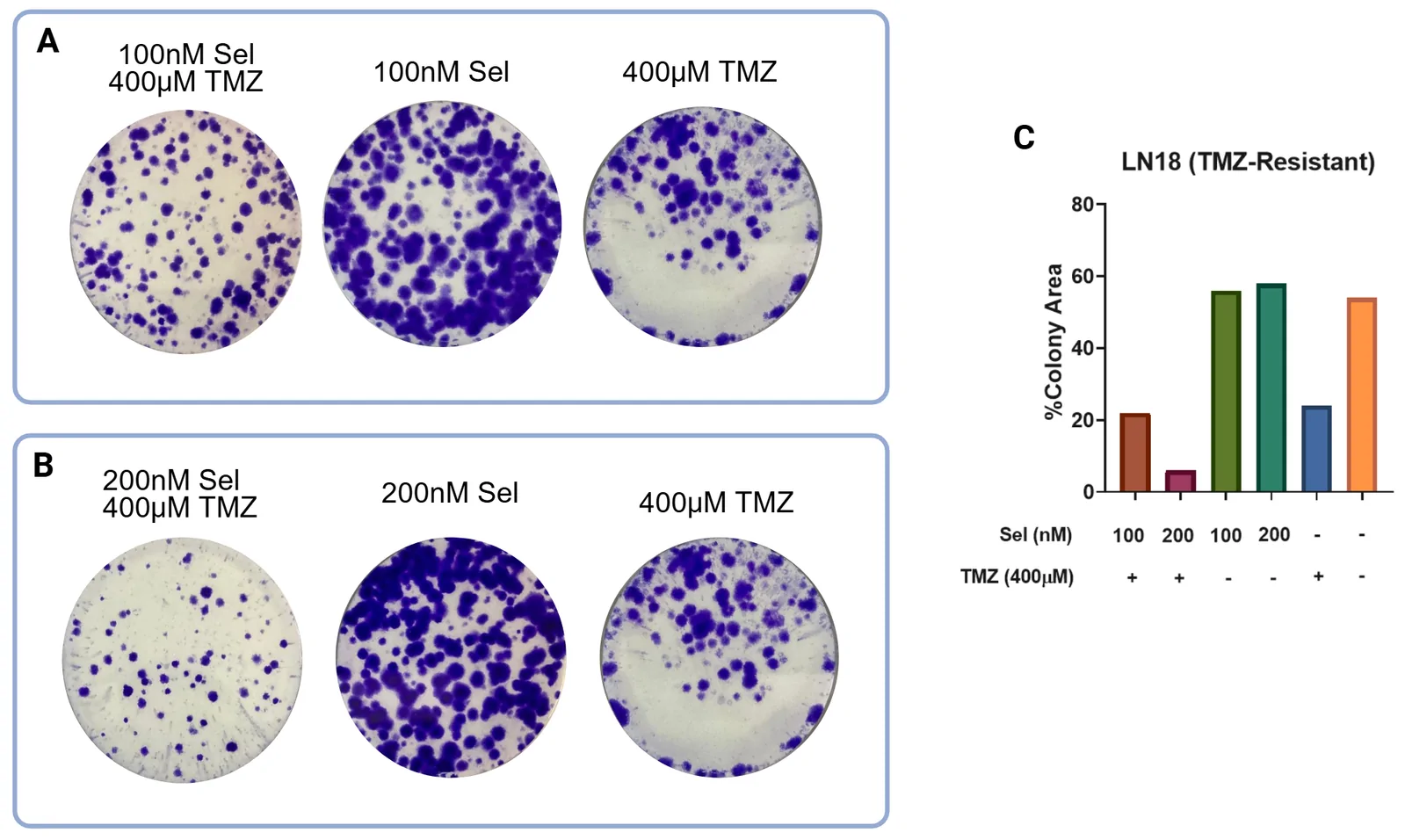
The selinexor and TMZ combination suppressed the long-term proliferative capacity of TMZ-resistant LN18 cells. **(A, B)** Representative crystal violet-stained colony formation images of LN18 cells treated with 100 nM selinexor + 400 µM TMZ combination, 100 nM selinexor alone, and 400 µM TMZ alone **(A)**, and 200 nM selinexor + 400 µM TMZ combination, 200 nM selinexor alone, and 400 µM TMZ alone **(B)**. **(C)** Quantification of colony area (%) for all treatment groups, including the untreated control. The colony area was determined using image analysis. Neither single agent meaningfully reduced colony formation below untreated levels, whereas combination treatment suppressed the colony area in a dose-dependent manner. The results represent a single independent experiment.

### 3.6 Nuclear p53 accumulation drives apoptotic protein signaling in TMZ resistant cells

Given that XPO1 mediates the nuclear export of p53, nuclear p53 localization was assessed by immunocytochemistry following treatment. Combination-treated LN18 cells exhibited markedly increased nuclear p53 accumulation compared with untreated and single-agent groups (**Figure 7**).

**Figure 7:**
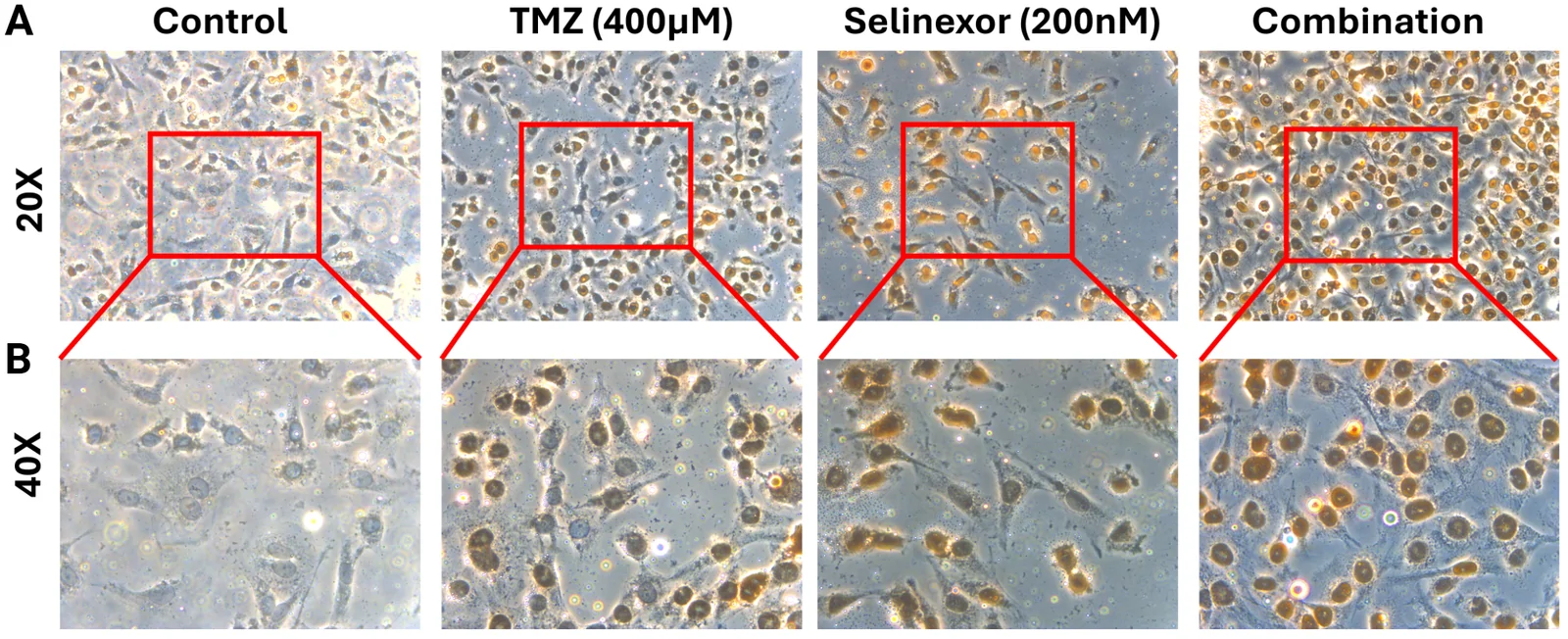
Selinexor and TMZ combination induced nuclear p53 accumulation in TMZ-resistant LN18 cells. **(A, B)** Representative immunocytochemistry images of LN18 cells treated with 400 µM TMZ alone, 200 nM selinexor alone, the combination, or left untreated for 24 h, shown at 20X **(A)** and 40X **(B)** magnification. The 40X images correspond to the boxed regions in **(A)**. Cells were fixed in ice-cold methanol, incubated with anti-p53 antibody, and visualized using an HRP-polymer detection system with AEC chromogen (brown) and hematoxylin counterstain (blue). Representative images show increased nuclear p53 immunoreactivity in combination-treated cells relative to untreated and single-agent controls.

To examine the downstream apoptotic response at the protein level, Western blot analysis of PARP, Caspase-9, and Bcl-2 was performed, with all values expressed as fold-change relative to untreated controls. Cleaved PARP reached 2.42-fold under combination treatment compared with 1.94-fold under TMZ alone, corresponding to a 24% greater increase, while full-length PARP was reduced to 0.64-fold under combination treatment versus 2.08-fold under TMZ alone, a 69% lower level (**Figure 8A**). Full-length Caspase-9 declined to 0.52-fold under combination treatment compared with 2.01-fold under TMZ alone, a 74% lower level (**Figure 8B**). Bcl-2 showed the most pronounced combination-specific effect, falling to 0.05-fold under combination treatment compared with 0.94-fold under TMZ alone, a 95% lower level, with TMZ alone remaining close to the untreated baseline (**Figure 8C**). Notably, TMZ alone increased rather than decreased full-length PARP and Caspase-9 levels, indicating that the loss of these proteins was specific to the combination rather than a general consequence of TMZ exposure.

**Figure 8:**
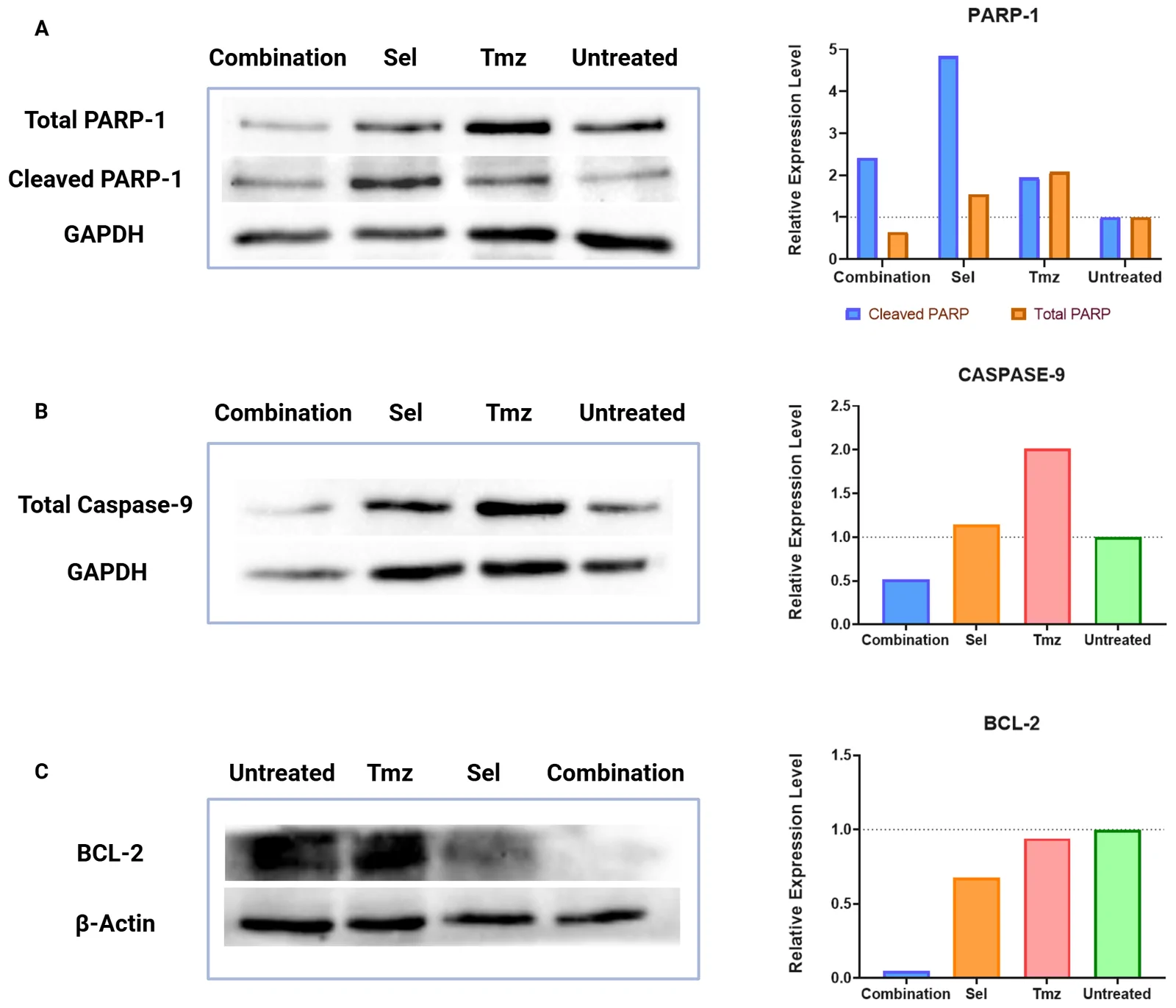
Selinexor and TMZ combination altered apoptotic protein levels in TMZ-resistant LN18 cells. **(A-C)** Representative Western blots and corresponding densitometric quantification for total and cleaved PARP-1 **(A)**, total caspase-9 **(B)**, and BCL-2 **(C)** in LN18 cells treated with 400 µM TMZ alone, 200 nM selinexor alone, the combination, or left untreated for 48 h. GAPDH was used as the loading control for PARP-1 and caspase-9, and β-actin for BCL-2. Each target protein was probed on a separate membrane with its corresponding loading control. Band intensities were quantified using ImageJ software, normalized to the respective loading control, and expressed as fold-change relative to the untreated control (dashed line, set at 1.0). Data are presented from a single independent experiment.

## 4 Discussion

Glioblastoma remains one of the most therapeutically intractable malignancies, with temozolomide (TMZ) resistance representing a central barrier to durable treatment response. Despite decades of research, the mechanisms underlying TMZ resistance are multifactorial and incompletely understood, encompassing MMR pathway dysfunction, epigenetic silencing of DNA damage response genes, and activation of compensatory survival signaling pathways (4, 43). Therefore, identifying agents capable of overcoming resistance without substantially increasing toxicity remains a critical unmet need. The present study demonstrates that selinexor, a first-in-class XPO1 inhibitor, sensitizes GBM cells to TMZ in a concentration-dependent manner, with the most clinically meaningful effects observed in the TMZ-resistant LN18 line. Collectively, our findings support selinexor as a mechanistically rational TMZ sensitizer in resistant GBM, acting through the convergent suppression of apoptotic escape, partial restoration of MMR-mediated damage recognition, and activation of DNA damage checkpoints.

The dose-response and viability matrix data established that selinexor was active at nanomolar concentrations across all three GBM lines, consistent with previous reports of XPO1 inhibitor efficacy in brain tumor models (44). Importantly, the selinexor concentrations used here (50–200 nM) fall within the range measured in human glioblastoma tissue: in a phase II study of oral selinexor in recurrent glioblastoma, median intratumoral drug concentration in resected tumors was 105.4 nmol/L (range 39.7–291 nmol/L), confirming that selinexor crosses the blood–brain barrier at pharmacologically active levels (45). Critically, the combination produced growth inhibition that exceeded that of either single agent across a broad dose range, an effect particularly pronounced in LN18 cells, where TMZ alone exerts minimal cytotoxic activity. The sensitization observed in LN18 represents a meaningful conversion of a TMZ-unresponsive line into one that responds to combination treatment, corroborated by ZIP synergy scores that were highest at intermediate selinexor doses, precisely the range where TMZ monotherapy fails. This sensitizing capacity is not restricted to alkylating agents: selinexor has been reported to enhance glioma cell sensitivity to other therapeutic agents (38, 39) and to increase radiosensitivity both *in vitro* and *in vivo* (41), indicating that XPO1 inhibition lowers the threshold for cell death across mechanistically distinct DNA-damaging modalities. Our data place TMZ within this group and further show that the effect is most pronounced where monotherapy resistance is greatest.

In TMZ-resistant LN18 cells, neither agent alone induced substantial apoptosis; however, their combination significantly exceeded both monotherapies, which is the defining hallmark of true sensitization rather than simple additivity. Delta apoptosis analysis further confirmed that both agents contributed independently and proportionally to this outcome, ruling out a scenario in which one drug drives the effect while the other is a bystander. This finding aligns with previous studies that showed selinexor induces apoptosis in GBM (46).

Gene expression data provide insight into the cooperative apoptotic effect. In LN18 cells, each single agent triggered a compensatory upregulation of MCL1 and BIRC6, anti-apoptotic proteins linked to therapy resistance in GBM and other malignancies (47, 48), whereas the combination uniquely suppressed this induction, removing the survival buffer that enables resistant cells to escape from cell death. This dual suppression, achieved by neither agent independently, provides a molecular basis for apoptotic induction in LN18 cells and suggests anti-survival signaling as a primary driver of selinexor-mediated TMZ sensitization. Moreover, the suppression of MCL1 by XPO1 inhibition is consistent with prior evidence that nuclear-retained pro-apoptotic factors directly counteract MCL1-mediated survival signaling (49). Protein-level analysis supported this transcriptional pattern. Bcl-2, another key anti-apoptotic regulator, was almost completely abolished under combination treatment while remaining near baseline with TMZ alone, mirroring the transcript-level suppression of MCL1 and BIRC6 and indicating that the combination dismantles anti-apoptotic buffering at both the transcriptional and protein levels. This is relevant in light of work showing that dual inhibition of XPO1 and Bcl-2/Bcl-xL has promising activity in GBM model systems (40); the near-complete loss of Bcl-2 observed here with selinexor and TMZ suggests that comparable anti-apoptotic disarmament may be reached without adding a second targeted agent, a consideration with direct implications for tolerability in a patient population already limited by treatment burden.

Consistent with the loss of this survival buffer, PARP cleavage was increased under combination treatment relative to TMZ alone, accompanied by a corresponding decrease in full-length PARP. Full-length Caspase-9 was likewise reduced under combination treatment but not with TMZ alone, providing additional support for this interpretation, although the absence of cleaved fragment detection precludes direct confirmation of caspase-9 processing. The concurrent nuclear accumulation of p53 observed by immunocytochemistry provides the mechanistic basis for this shift, in agreement with previous reports that selinexor-mediated XPO1 blockade promotes nuclear p53 retention in solid tumor models (32, 50), and links restored p53 function to the apoptotic protein changes described above. In contrast, LN229 cells showed smaller delta apoptosis values and limited combination-single agent separation, consistent with a ceiling effect, where both agents are already individually active, reinforcing that the combination’s apoptotic benefit is most meaningful in the resistant context.

Cell cycle data revealed distinct line-specific redistribution patterns consistent with differential checkpoint engagement. In LN18 cells, an inverse dose-dependent shift from S phase to G2/M was observed exclusively in the combination groups, suggesting that the combination drives checkpoint activation in a way that neither agent achieves alone. This finding is further supported by CHK1 upregulation in these same treatment groups and is consistent with prior evidence that selinexor facilitates the nuclear accumulation of checkpoint regulators, including CHK1 (51). Cells arrested at G2/M under conditions of unrepaired alkylation damage and suppressed MCL1 signaling, driving them toward apoptosis rather than checkpoint recovery. In U87MG cells, S phase accumulation was the predominant combination-specific effect, consistent with impaired replication fork progression under potentiated DNA damage (52). In LN229 cells, G2/M elevation was uniform across all TMZ-containing groups, reflecting the expected checkpoint response of an intrinsically TMZ-sensitive line, where monotherapy alone is sufficient to engage the damage response consistent with CHK1 upregulation.

The colony formation data provided functional confirmation of the short-term viability and apoptosis findings in a longer-term context. The near-complete suppression of colony-forming capacity at 200 nM selinexor combined with 400 µM TMZ in LN18 cells, while neither single agent meaningfully reduced colony growth, represents the most direct functional evidence of resistance reversal in this study. Colony formation assays capture a distinct biological endpoint from acute viability or apoptosis measurements, reflecting the ability of surviving cells to re-establish proliferative growth following treatment (53). The inability of combination-treated LN18 cells to do so, even at clinically relevant selinexor concentrations, indicates that the combination does not merely delay growth but durably undermines the proliferative capacity of resistant cells, which is an important translational consideration, as sustained tumor control rather than transient growth inhibition remains the primary clinical objective in GBM.

These findings can be positioned against other efforts to reverse TMZ resistance, and one comparison requires particular attention. Mapunda et al. reported that selinexor-TMZ sensitization was confined to MGMT-methylated GBM lines and that forced MGMT expression abolished it, concluding that selinexor may enhance MGMT-dependent TMZ resistance through PKA-CREB-mediated MGMT induction (42). Among the lines used here, LN18 carries an unmethylated MGMT promoter and expresses functional MGMT (54), whereas U87MG and LN229 carry methylated promoters with silenced MGMT (55, 56). On the basis of that model, MGMT-proficient LN18 would not be expected to be sensitized, yet it showed the strongest sensitization among the three lines examined.

We propose that this reflects a second, MGMT-independent axis of resistance that the combination engages. TMZ resistance persists in a substantial fraction of MGMT-methylated tumors (8), MMR dysfunction is an established resistance determinant independent of MGMT status (10, 11), and MGMT-independent resistance mechanisms have been described directly in glioblastoma cells (57). Relevant to our lines, an inverse relationship between MGMT and MMR expression has been reported in GBM cell lines, with high MGMT-expressing, TMZ-resistant lines lacking MMR complex expression and MGMT-silenced, TMZ-sensitive lines showing high MMR expression (56). Our gene expression data recapitulate this pattern: MMR transcripts remained at or below baseline across all treatment groups in MGMT-proficient LN18 cells, whereas MGMT-silenced LN229 cells showed constitutive MMR engagement. Critically, in LN18 cells the combination raised MMR expression above the level reached by either single agent, alongside the loss of anti-apoptotic signaling described above. This suggests that selinexor sensitizes LN18 cells not through the MGMT axis but by partially restoring damage recognition and removing apoptotic escape, mechanisms that remain available irrespective of MGMT status. As MGMT protein and activity were not measured here, whether the MGMT-inducing effect reported by Mapunda et al. offsets this benefit in other MGMT-proficient backgrounds remains to be determined.

A separate strategy, PARP inhibition, restores TMZ sensitivity independently of MGMT status by targeting base excision repair directly (15). The mechanism identified here acts instead on damage recognition and apoptotic commitment, indicating that these approaches address distinct nodes of the same resistance problem and may prove complementary. Establishing whether selinexor-mediated sensitization holds across defined MGMT and MMR backgrounds, and whether it translates to *in vivo* and patient-derived models, will determine where this combination fits among emerging resistance-reversal strategies.

## 5 Limitations

This study has several limitations. All experiments were conducted *in vitro* using established GBM cell lines, which may diverge from the tumors of origin and do not reproduce intratumoral heterogeneity or blood-brain barrier pharmacokinetics; validation in patient-derived cells and orthotopic *in vivo* models remains necessary. XPO1 inhibition was inferred from nuclear p53 accumulation rather than demonstrated by genetic perturbation, and MGMT protein levels were not measured. Western blot analyses were limited to LN18 cells and to a single biological experiment, so the reported fold-changes are descriptive; the cleaved fragments of caspase-3 and caspase-9 were not reliably detected, and engagement of the intrinsic apoptotic pathway therefore rests on PARP cleavage and reduced full-length caspase-9. Gene expression data were similarly interpreted as directional trends given inter-experimental variability.

## Data Availability

The raw data supporting the conclusions of this article will be made available by the authors, without undue reservation.
