# Supplementary material for: Selinexor enhances temozolomide efficacy in glioblastoma through modulation of multiple resistance-associated mechanisms
Source: Front Oncol. 2026 Sep 28;16:1916260. doi: 10.3389/fonc.2026.1916260 (PMC13655233; doi:10.3389/fonc.2026.1916260)
Supplement: Supplementary file 1 [file SupplementaryFile1.docx]

Supplementary Material

# Supplementary Figures

**Supplementary Figure 1.** Statistical validation of selinexor and TMZ combination treatment on cell viability across GBM cell lines. **(A)** LN18 (TMZ-resistant), **(B)** U87MG (TMZ-moderate), and **(C)** LN229 (TMZ-sensitive) cells treated with the indicated selinexor and TMZ dose combinations at 48 h and 72 h. Bar charts show cell viability (%) for combination (Sel+TMZ), selinexor alone (Sel), and TMZ alone (TMZ) groups. Only dose and time point combinations that reached statistical significance are shown. Data are presented as mean ± SD of triplicate measurements from a representative experiment. Statistical significance was determined by one-way ANOVA with Holm–Šídák multiple comparisons test; all comparisons shown are between the combination treatment group and the corresponding single agent groups. ****p<0.0001, ***p<0.001, **p<0.01, *p<0.05, ns = not significant.

**Supplementary Figure 2.** Percentage apoptosis in LN18 (TMZ-resistant) cells treated with escalating Selinexor concentrations (50, 100, 200 nM) combined with a fixed TMZ dose of 200 µM, compared to single agent and untreated controls. Data are presented as mean ± SD of triplicate measurements from a representative experiment. Statistical significance was determined by one-way ANOVA with Holm–Šídák multiple comparisons test; all comparisons shown are between the combination treatment group and the corresponding single agent or untreated control groups. ***p<0.001, **p<0.01, *p<0.05, ns = not significant.
